# Optimizing vestibular neuritis management with modular strategies

**DOI:** 10.3389/fneur.2023.1243034

**Published:** 2023-09-14

**Authors:** Fei Li, Jin Xu, Dan Liu, Jun Wang, Lingmei Lu, Rui Gao, Xiaowen Zhou, Jianhua Zhuang, Sulin Zhang

**Affiliations:** ^1^Department of Neurology, Second Affiliated Hospital of Naval Medical University, Shanghai, China; ^2^Department of Otorhinolaryngology, Union Hospital, Tongji Medical College, Huazhong University of Science and Technology, Wuhan, China; ^3^Institute of Otorhinolaryngology, Union Hospital, Tongji Medical College, Huazhong University of Science and Technology, Wuhan, China; ^4^Department of Neurology, Qidong People's Hospital, Qidong Liver Cancer Institute, Affiliated Qidong Hospital of Nantong University, Qidong, China

**Keywords:** vestibular neuritis, modular management, risk factor, intervention, prognosis

## Abstract

**Objective:**

This study proposes a “modular management” approach for vestibular neuritis (VN) to reduce chronicization and improve patient prognosis. The approach involves multi-factor grading and hierarchical intervention and was found to be more effective than traditional treatment strategies.

**Methods:**

This retrospective analysis compared two groups of VN patients from two medical institutions. The intervention group of 52 patients received “modular management,” while the control group of 51 patients did not receive this kind of management. Analyzed the early treatment strategies, 6-month prognosis, and other indicators of the two groups of patients, compared and analyzed their overall prognosis, and identified the risk factors affecting the chronicization.

**Results:**

The modular management group had lower dizziness severity, better balance, lower anxiety, and higher video head impulse testing (v-HIT) gain after 6 months of onset. Analysis of factors related to persistent postural-perceptual dizziness (PPPD) in patients with VN showed positive correlations between the time from onset to diagnosis and PPPD, and Vertigo Symptom Scale (VSS), Dizziness Handicap Inventory (DHI), anxiety, and depression. Normalized vestibular rehabilitation was negatively correlated with PPPD, while gender, age, and early steroid use had no significant correlation. The multi-factor logistic regression model correctly classified 93.20% of the study subjects with a sensitivity of 87.50% and specificity of 94.90%.

**Conclusion:**

The proposed “modular management” scheme for VN is a comprehensive and dynamic approach that includes health education, assessment, rehabilitation, therapy, evaluation, and prevention. It can significantly improve patient prognosis and reduce chronicization by shifting from simple acute treatment to continuous management.

## 1. Introduction

Vestibular neuritis (VN) refers to an acute impairment of unilateral peripheral vestibular function, which is clinically characterized by acute and persistent vertigo (1), accompanied by nausea, vomiting, unsteadiness, and a tendency to fall toward the affected side. It is a common acute vertigo syndrome in clinical practice (2) VN has a certain degree of spontaneous recovery, and in clinical practice, the prognosis of most VN patients is good with low recurrence rates. However, some patients may develop chronic symptoms. Previous studies on VN patients have found that 25–50% of patients develop persistent postural-perceptual dizziness (PPPD) during 3–12 months of follow-up (3, 4), around 15–30% of VN patients still experience persistent dizziness and oscillopsia 1 year after the onset of the disease (5). Once VN evolves into PPPD, patients will suffer from chronic and persistent dizziness/unsteadiness that is exacerbated by an upright posture, movement, or visual stimuli (6, 7). The development of secondary functional disorders, such as gait changes, anxiety, avoidance behavior, increased heart rate, and sweating, is also observed in patients with PPPD (8, 9). Chronic symptoms of PPPD can lead to significant decline in social functioning and some patients may become housebound and lose the ability to engage in daily activities and work. Therefore, effective prevention of VN chronicization is crucial for long-term prognosis and the patient’s ability to return to normal social life.

Research on risk factors related to the chronicization of VN has not yielded a consistent conclusion. Kim et al. (10) suggested that the gain of semicircular canal in video head impulse testing (v-HIT) is the best predictor of patient symptom recovery, and a decrease in canal gain (<0.5) often indicates a prolonged disease course. Patients with covert saccades showed relatively better recovery of dynamic visual acuity, gait, and balance compared to those with overt saccades (11). However, a study by Patel et al. (12) suggested that there is no correlation between the chronicity of VN in patients and the results of bithermal caloric testing or v-HIT. Furthermore, previous studies have suggested that the chronicity of VN is associated with factors, such as patient anxiety status, personality traits, and visual dependence (13–19). Therefore, it is currently believed that the prognosis of VN patients may be related to multiple factors, and the weight of each factor may vary among individual patients. However, previous studies have mainly focused on analyzing the correlation between clinical characteristics during the acute and/or recovery periods and prognosis, but rarely addressed how to develop reasonable treatment plans to improve patient prognosis. So far, there is a lack of clear guidance on when and how to intervene, as well as how to evaluate the effectiveness of interventions for chronic risk factors such as residual vestibular dysfunction, anxiety and depression, and visual dependence. Clinical physicians often rely on personal experience when diagnosing and treating patients with VN. In our past clinical work, we have found that some doctors tend to prioritize medication over explaining the disease, and focus more on conducting tests rather than providing rehabilitation guidance. These practices leading to the chronicization of VN. Therefore, we believe that differences in clinical concepts may be an important factor contributing to differences in the prognosis of VN. Therefore, standardizing clinical diagnosis and treatment plans can help reduce the proportion of patients who develop chronic conditions, save medical expenses, and promote overall recovery.

This study proposes a modular management approach for VN, which shifts from simple acute-phase diagnosis and treatment to continuous, comprehensive, and dynamic management that includes disease knowledge dissemination, vestibular function assessment, vestibular rehabilitation (VR), cognitive behavioral therapy (CBT), prognosis evaluation, and prevention of chronicization. This study aims to investigate whether the “modular management” approach can improve the prognosis, reduce chronicization, and promote rapid recovery of social function in VN patients.

## 2. Materials and methods

### 2.1. Modular management for VN

#### 2.1.1. Modular management for VN (acute phase)

##### 2.1.1.1. Applicable patients

Patients with acute onset vertigo within 14 days, or with spontaneous nystagmus (SN) to the healthy side on bedside examination.

##### 2.1.1.2. Bedside assessment

Perform a comprehensive physical examination of neurology and otology to differentiate and establish a definitive diagnosis. The evaluation should include the following components: ① Spontaneous nystagmus (SN), gaze-evoked nystagmus, and fixation suppression (horizontal head shaking or hyperventilation can be used based on patient tolerance); ② Bedside horizontal head impulse test and ocular tilt reaction (OTR); ③ Romberg test, tandem gait test, and finger-to-nose test; and ④ Tuning fork hearing test and otoscopy.

##### 2.1.1.3. Auxiliary examination

1. The type and timing of vestibular function tests should be based on the patient’s tolerance. When a patient is in generally well health to tolerate vestibular function tests, these tests should be conducted as early as possible. Such tests are beneficial for accurate diagnosis, individualized vestibular rehabilitation (VR) program development, and prognosis evaluation.

The required tests include: ① Video nystagmography and caloric testing; ② Video head impulse test (v-HIT).

Optional tests include: ① Vestibular-evoked myogenic potential testing (VEMP); ② Fundus photography and ocular tilt reaction testing; and ③ Mid-frequency rotation.

2. Complete cranial MRI and pure tone audiometry (PTA) testing to rule out central structural lesions or otologic disorders. If necessary, other imaging tests such as CT plain or enhanced of the inner ear.

##### 2.1.1.4. Treatment of acute phase patients

1. Symptomatic support: In the initial 48 h of VN onset, patients may experience severe dizziness, nausea, and vomiting. Symptomatic relief with vestibular suppressants is important to prevent complications, but prolonged use can hinder central compensation (20).
2. Glucocorticoid: For patients without contraindications to steroid use, short-term low-dose corticosteroid therapy can be given during the acute phase. The specific regimen is as follows: prednisone 1 mg/kg/day for 5 consecutive days, followed by tapering to 0.5 mg/kg/day for 3 consecutive days.
3. VRT: To facilitate clinical practice, we have divided the rehabilitation program for the acute phase of VN into two stages based on the characteristics of the disease course. Details below.

###### 2.1.1.4.1. Stage A

- Seated oculomotor training (for patients whose static compensation has not been fully completed and who still have SN). ① The exercise involves fixing gaze on a visual target (in the forward and lateral directions) and maintaining eye position, followed by closing the eyes for 2–3 s and reopening them to continue gazing at the same target; ② Visual tracking exercises; and ③ Eye scanning exercises.
- Postural exercises (for patients whose static compensation has been mostly completed and whose SN has disappeared or significantly weakened). ① Standing eyes-open and eyes-closed training; ② Weight shift training; ③ Gait training; ④ Single-leg standing training; and ⑤ Tandem standing training.

###### 2.1.1.4.2. Stage B

(For patients who have completed the acute phase training program) ① Standing, walking, and turning without visual cues or with altered proprioceptive feedback (such as foam pads or moving platforms) present; ② Gaze stability training (VOR × 1, VOR × 2) (21); ③ The vestibular substitution gaze stabilization training typically involves a duration of 12 min per session during the acute phase, with a minimum of three sessions per day. The final rehabilitation plan should be developed collaboratively by a vestibular specialist and rehabilitation therapist, with dynamic adjustments based on the individual needs of the patient.

4. Patients’ education: Dizziness specialists should educate patients and their families about the illness characteristics and prognosis from consultation to complete recovery. The communication should focus on the underlying reasons for dizziness, recovery principles, and benign prognosis. Various forms of communication, such as verbal, written, video, group counseling, and individual Q&A sessions, can be used.
5. Promote VC drug: Recommend using drugs that promote vestibular compensation (VC), such as EGb761, an extract of Ginkgo biloba (80 mg orally three times a day for 3 months) and betahistine dihydrochloride (22) (12 mg orally three times a day for 3 months).

### 2.2. Modular management for VN (recovery phase)

#### 2.2.1. Applicable patients

Acute dizziness with an onset between 14 days and 3 months ago, and no SN detected by bedside examination.

#### 2.2.2. Bedside assessment

The same as acute phase.

#### 2.2.3. Auxiliary examination

① The same as acute phase; ② For patients without a clear history of AVS, further investigations should be conducted to exclude EVS or CVS.

#### 2.2.4. Treatment of recovery phase patients

**Figure 1:**
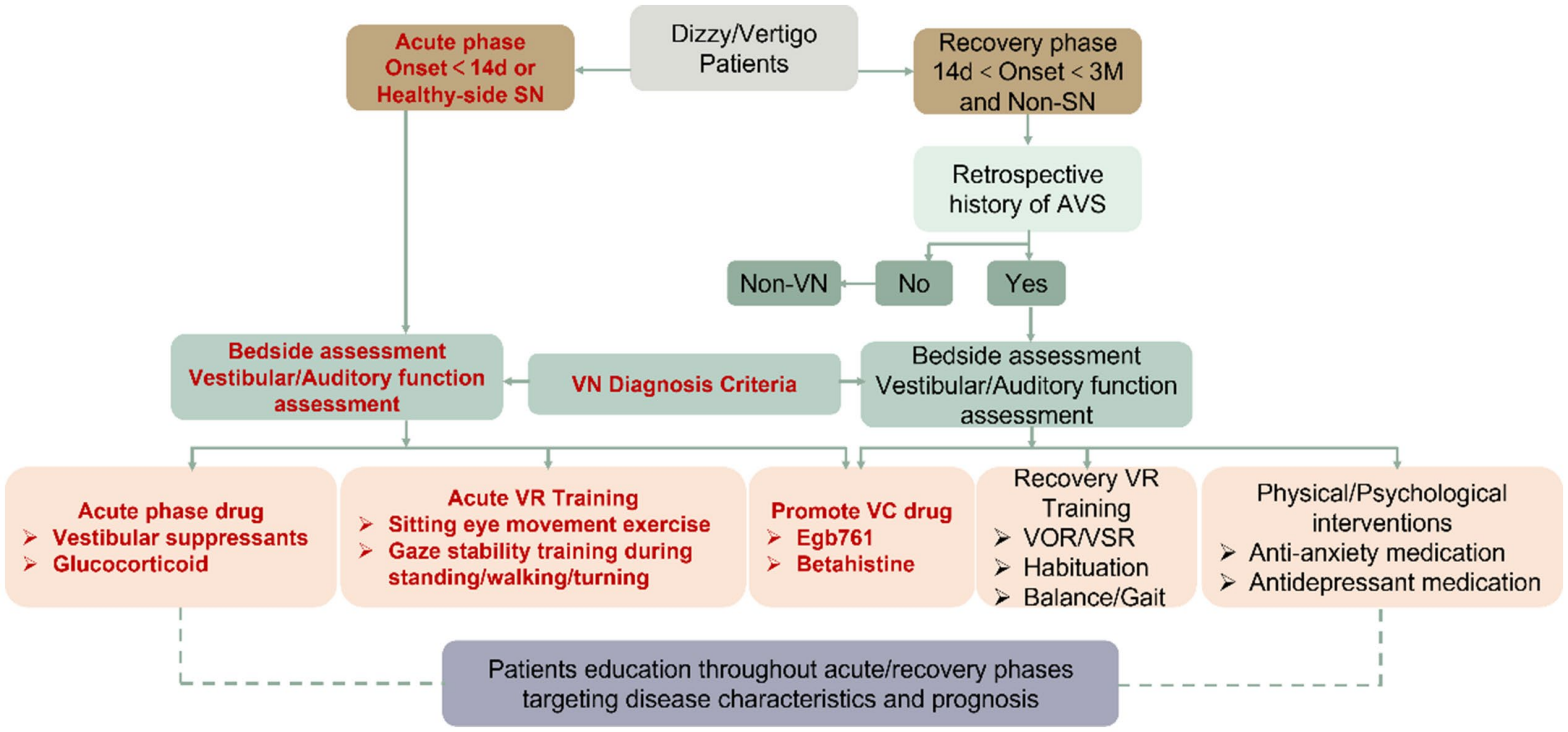
Modular management flowchart for VN. The flowchart presented in this paper represents a logical sequence of modules that can be used to manage vestibular neuritis effectively. The design incorporates key components such as vestibular rehabilitation, pharmacological interventions, lifestyle modifications, and psychological support. Each module is tailored to address specific needs and challenges encountered during different stages of the condition. VN, Vestibular neuritis; SN, Spontaneous nystagmus; VR, Vestibular rehabilitation; AVS, Acute vestibular syndrome; and VC, Vestibular compensation.

During recovery, if patients lack proper diagnosis and treatment in the acute phase, they may restrict movements to alleviate discomfort. However, this can hinder muscle function and limit social activity. Educating and encouraging patients to increase movement and exposure is vital for recovery. To develop a personalized VR plan, consider the patient’s complaints, clinical manifestations, and vestibular function tests. Adjust training intensity gradually, supervise therapy or offer home options, start with simple exercises, and modify the plan regularly. Early VR exercise initiation promotes faster and more complete vestibular function recovery (23, 24) (Figure 1).

1. This rehabilitation program is for the pre-recovery phase of vestibular disorders for patients who can complete acute phase training. ① Cross-coupled tracking: Hold a playing card in each hand and cross-move them while keeping the eyes fixed on one of the cards throughout the exercise; ② Postural control training; ③ Stair climbing exercise; ④ Walking while performing left–right gaze shifts, walking while performing up-down gaze shifts, and multitasking while walking; ⑤ Anti-saccade training, memory-based vestibulo-ocular reflex (VOR) training, memory-based saccade training; ⑥ Turning exercise; ⑦ Obstacle avoidance training; ⑧ Complex background gaze training; and ⑨ Endurance training.
2. Promote VC drug: The same as acute phase.
3. Thoroughly evaluate anxiety and depression levels in patients. Use cognitive behavioral therapy (CBT) for mild cases and anti-anxiety/antidepressant treatments for moderate to severe cases to increase compliance with rehabilitation. The 14-item Hospital Anxiety and Depression Scale (HADS), Vertigo Symptom Scale (VSS), and Dizziness Handicap Inventory (DHI) are helpful tools for clinicians to objectively assess the physical and mental conditions of patients.

#### 2.2.5. Patients and inclusion/exclusion criteria

Inclusion criteria for the modular management group were: ① patients with VN treated in vertigo specialty clinics at Shanghai Changzheng Hospital and Wuhan Union Hospital between January 2019 and June 2021. The diagnosis of acute VN was based on a history of sudden onset of vertigo, without auditory or neurological symptoms. The clinical findings comprised of spontaneous contralesional horizontal-torsional nystagmus that did not change direction with gaze and increased without visual fixation and an ipsilesional pathologic head impulse test (25), it also complies with the diagnostic criteria for Acute Unilateral Vestibulopathy (AUVP) (26). ② We included patients who met the criteria for the “modular management program for vestibular neuritis” and had complete clinical case data. All patients were followed up for at least 6 months after their visit (follow-up methods included in-person visits and telephone follow-up).

The control group comprised patients diagnosed with VN at Shanghai Changzheng Hospital and Wuhan Union Hospital or in a vertigo specialty clinic within 1–6 months after onset, excluding those in the modular management program. Telephone interviews were conducted for follow-up assessments.

This study was approved by the Ethics Committee of Union Hospital, Tongji Medical College, Huazhong University of Science and Technology, Wuhan, China (NO. 20210873). All procedures performed in the studies involving human participants were in strict accordance with the ethical standards of the institutional and/or national research committee and with the 1964 Helsinki Declaration and its later amendments or comparable ethical standards.

### 2.3. Methods

#### 2.3.1. Rating scale

1. The 14-item Hospital Anxiety and Depression Scale (HADS) (27) has four response categories (0–3). Total score ranges from 0 to 42 points. A score of 19 points or more indicates a case of anxiety or depression, whereas 15 points indicate a possible case.
2. Vertigo Symptom Scale (VSS) (28). The scale has five response categories (0–4). Total scale scores range between 0 and 60 points, the scale has five response categories (0–4). Total scale scores range between 0 and 60 points，severe dizziness ≥ 12 points, clinically significant change ≥ 3 points.
3. Dizziness Handicap Inventory (DHI) (29) which has three response categories (0, 2, and 4). Total scores range from 0 to 100 points, interpreted as mild 0–30; moderate 31–60; and severe 61–100.

#### 2.3.2. Vestibular function test

Patients in the modular management group have vestibular function tests 1 week after diagnosis and at 6 months after onset. Non-modular group (control group) patients have tests at 6 months after onset upon study entry. Tests are performed by technicians with at least 3 years of experience.

##### 2.3.2.1. Caloric test

Subject lies flat with head elevated by a 30° pillow. Eye movement recording system measures SPV, CP, and DP. Air irrigation at 24 and 50°C with 5 L/min for 60 s (30).

##### 2.3.2.2. V-HIT

Video head impulse testing conducted using videonystagmography system with subjects positioned 1.2 m from eye-level target. Goggles secured with elastic band. Multiple rotations performed on each side (31).

#### 2.3.3. Diagnostic criteria of PPPD

Diagnostic criteria for PPPD: (1) Persistent dizziness/unsteadiness lasting ≥3 months. (2) Symptoms during upright position or triggering situations. (3) Not explained by other conditions. (4) Not accounted for by psychiatric disorders. (5) Causes distress/impairment. Diagnosis requires all criteria and excludes other conditions (3, 8).

### 2.4. Statistical analysis

In SPSS 23.0 software, normality and homogeneity of variance were tested before comparing continuous data. Normally distributed data were expressed as $\bar{x}$ ± *s* and compared using a *t*-test if meeting the assumptions; otherwise, the Mann–Whitney U test was used. Categorical data were presented as *n* and %, and compared using *χ*^2^ test or Fisher’s test. Kendall’s tau-b correlation and multivariate logistic regression analysis were performed to establish a regression model and evaluate multicollinearity. The ROC curve was used to analyze the predictive value of each indicator for PPPD development. The significance level was set at 0.05.

## 3. Results

**Figure 2:**
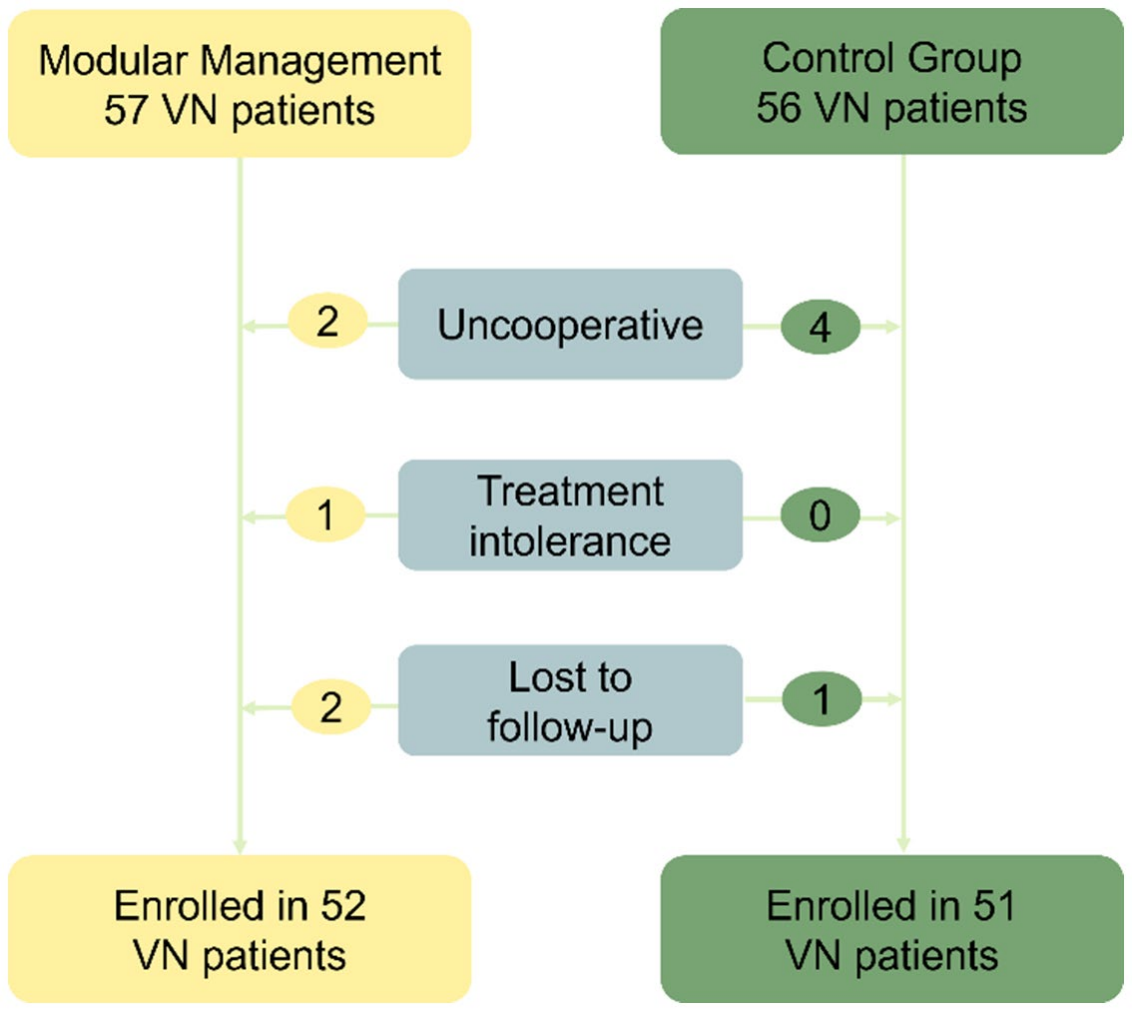
Flowchart for the screening process. The study included a total of 103 patients, with 57 patients in the modular management group. However, two patients were uncooperative during examinations, one patient was intolerant to treatment, and two patients were lost to follow-up. As a result, 52 patients were ultimately included. In the control group, 56 patients were followed up, but four were uncooperative during examinations and one was lost to follow-up, leading to 51 patients being included. VN, Vestibular neuritis.

In the modular management group, 57 patients were followed up, but two patients were uncooperative during examinations, one patient was intolerant to treatment, and two patients were lost to follow-up. Therefore, 52 patients were included in the study. In the control group, 56 patients were followed up, but four were uncooperative during examinations and one was lost to follow-up, resulting in 51 patients being included in the study. Overall, 103 patients were included in the study (Figure 2). Average age: 54.22 ± 12.899 years (15–82 years). Onset to diagnosis time: 16 (14–28) days. Onset to remission time: 6 (4–7) days. Male cases: 51, Female cases: 52, Male-to-female ratio: 1:1.020.

### 3.1. Baseline comparison between modular management and control groups

Baseline comparison showed no significant differences (*p* > 0.05) between the two groups in age, gender, time of visit, and various scale parameters during the early stage of the disease (Table 1).

**Table 1:** Baseline comparison between modular management and control groups.

Parameter	Control group	Modular management	Statistical value	*p* value
	*n* = 51	*n* = 52		
Age (year)	53.59 ± 11.979	54.85 ± 13.831	*F* = 0.243	0.623
Gender (*n*, %)			*χ*^2^ = 0.244; *p* = 0.622	
Male	24 (47.060)	27 (51.920)		
Female	27 (52.940)	25 (48.080)		
Onset-to-diagnosis (d)	15.000	16.670	*U* = 1098.000	0.131
Onset-to-relief (d)	5.670	6.320	*U* = 1177.000	0.321
VSS (score), *M*	55.000	58.330	*U* = 1258.000	0.654
DHI (score), *M*	54.200	54.600	*U* = 1246.000	0.597
ABC (%), *M*	50.863	50.450	*U* = 1259.500	0.660
HADS-A (score), *M*	4.000	4.820	*U* = 1300.000	0.863
HADS-D (score), *M*	1.590	1.610	*U* = 1304.500	0.884

VSS: Vertigo symptom scale; DHI, Dizziness handicap inventory; ABC, Activities-specific balance confidence scale; HADS, The 14-item hospital anxiety and depression scale. ^*^p < 0.05, ^**^p < 0.01, and ^***^p < 0.001.

### 3.2. Comparison of treatment and 6-month prognosis between modular management and control groups

We found that patients treated with modular management therapy were more likely to receive early corticosteroid treatment and had higher compliance with rehabilitation treatment. During the 6-month follow-up period, 10.68% of patients experienced recurrent dizziness, with most of them having a history of dizziness before VN onset. The incidence of PPPD differed significantly between the modular and non-modular management groups (*p* < 0.000). At 6 months after disease onset, all enrolled patients underwent reassessment of vestibular function, with the following parameters recorded: canal paresis (CP) values (normal range < 30%) and affected-side horizontal video head impulse test (vHIT) gains (normal range 0.8–1.2). In the modular management group, the parameters for the two groups at 6 months were as follows: 32 patients (61.538%) achieved normalization of CP values, 34 patients (65.385%) achieved normalization of vHIT gains, and 23 patients (44.231%) achieved normalization of both parameters. In contrast, in the non-modular management group, the respective numbers were 25 patients (49.020%), eight patients (15.686%), and four patients (7.843%). At 6 months after onset, the modular management group had better recovery of vHIT gain and lighter symptoms of dizziness, balance disorders, and anxiety than the non-modular group (Table 2).

**Table 2:** Comparison of treatment and prognosis between modular management and control groups.

Parameter	Control group (*n* = 51)	Modular management (*n* = 52)	Statistical value	*p* value
Low-dose steroid in acute phase (*n*, %)	6 (11.765)	35 (67.308)	*χ*^2^ = 33.151	0.000^***^
Standardized VRT (*n*, %)	7 (13.725)	52 (100.000)	*χ*^2^ = 78.320	0.000^***^
Incidence rate of PPPD (*n*, %)	20 (39.216)	4 (7.692)	*χ*^2^ = 14.317	0.000^***^
CP value (%), *M*	31.000	25.860	*U* = 1137.500	0.213
vHIT gain, *M*	0.660	0.824	*U* = 497.000	0.000^***^
VSS (score), *M*	7.000	3.400	*U* = 1023.000	0.045^*^
DHI (score), *M*	2.210	1.890	*U* = 1251.500	0.610
ABC (%), *M*	88.920	95.000	*U* = 796.000	0.000^***^
HADS-A (score), *M*	3.900	2.380	*U* = 916.000	0.006^**^
HADS-D (score), *M*	1.000	0.910	*U* = 1249.500	0.599

VRT, Vestibular rehabilitation therapy; CP, canal paresis; PPPD: Persistent postural-perceptual dizziness; v-HIT, video-Head impulse test; VSS, Vertigo symptom scale; DHI, Dizziness handicap inventory; ABC, Activities-specific balance confidence scale; and HADS, The 14-item hospital anxiety and depression scale. ^*^p < 0.05, ^**^p < 0.01, and ^***^p < 0.001.

### 3.3. Comparison of vestibular function and scale between two time points in modular management group

The vestibular function and various clinical parameters, including dizziness symptoms, social function, balance disorders, and anxiety, showed significant improvement in the modular management group compared to the non-modular group at both the early stages of the disease and 6 months after onset (*p* < 0.05; Table 3).

**Table 3:** Comparison of vestibular function and scale between two time points in modular management group.

Parameter	First onset	6 months post-onset	*U-v*alue	*p* value
CP value (%), *M*	50.830	25.860	409.000	0.000^***^
vHIT gain, *M*	0.552	0.824	197.500	0.000^***^
VSS, *M*	58.330	3.400	19.000	0.000^***^
DHI, *M*	54.600	1.890	0.000	0.000^***^
ABC (%), *M*	50.450	95.00	22.000	0.000^***^
HADS-A, *M*	4.820	2.380	793.000	0.000^***^
HADS-D, *M*	1.610	0.910	998.500	0.018^*^

CP, Canal paresis; vHIT, Video-head impulse test; VSS, Vertigo symptom scale; DHI, Dizziness handicap inventory; ABC, Activities-specific balance confidence scale; and HADS, The 14-item hospital anxiety and depression scale. ^*^p < 0.05, ^**^p < 0.01, ^***^p < 0.001.

### 3.4. Analysis of factors related to secondary PPPD in VN patients

A correlation analysis was conducted to explore the factors related to the development of PPPD in patients with VN. Results showed that patients with longer time from onset to diagnosis, higher VSS and DHI scores at 6 months after onset, and more severe anxiety and depression were more likely to develop PPPD. Patients who received standardized vestibular rehabilitation therapy had a significantly lower likelihood of developing PPPD (*p* < 0.05). There was no significant correlation between gender, age, or early use of steroids and the development of PPPD (Table 4).

**Table 4:** Univariate analysis of VN chronicization factors.

Parameter	Kendall’s tau-b	*p* value
	Correlation coeff	
Gender	−0.143	0.148
Age	−0.022	0.785
Onset-to-diagnosis	0.285	0.001^**^
Onset-to-relief	0.016	0.847
Initial assessment post-onset		
CP value	−0.090	0.439
vHIT value	0.150	0.197
VSS	0.073	0.376
DHI	0.046	0.582
ABC	0.017	0.842
HADS-A	0.096	0.255
HADS-D	0.159	0.071
Assessment 6 months post-onset		
CP value	0.135	0.100
vHIT value	−0.142	0.086
VSS	0.165	0.049^*^
DHI	0.495	0.000^***^
ABC	−0.147	0.077
HADS-A	0.558	0.000^***^
HADS-D	0.381	0.000^***^
Steroids early?	−0.167	0.092
Standard VR?	−0.453	0.000^***^

CP, Canal paresis; vHIT, Video-Head impulse test; VSS, Vertigo symptom scale; DHI, Dizziness handicap inventory; ABC, Activities-specific balance confidence scale; HADS, The 14-item hospital anxiety and depression scale; VR, Vestibular rehabilitation. ^*^p < 0.05, ^**^p < 0.01, ^***^p < 0.001.

We established a model using multiple logistic regression to identify independent factors associated with the development of PPPD in patients with VN. The model had statistical significance (*p* = 0.000) and accurately classified 93.200% of patients with a sensitivity of 87.50% and a specificity of 94.900%. There was no multicollinearity among the six predictor variables included in the model. The DHI score, anxiety level, and whether standard VRT was performed at 6 months after onset were identified as independent factors influencing the development of PPPD in patients with VN (Table 5).

**Table 5:** Multivariate logistic regression analysis of chronicization factors in VN.

Parameter	β	SE	OR	95% CI	*p* value
Onset-to-diagnosis (d)	0.024	0.018	1.024	0.998–1.062	0.195
Onset 6 months VSS	0.065	0.061	1.068	0.948–1.203	0.282
Onset 6 months DHI	0.387	0.186	1.473	1.023–2.120	0.037^*^
Onset 6 months HADS-A	0.716	0.262	2.047	1.226–3.419	0.006^**^
Onset 6 months HADS-D	0.330	0.246	1.391	0.859–2.254	0.180
Standard VR?	2.996	1.486	19.998	1.086–368.211	0.044^*^
Constant	−10.261	2.959	0.000	/	0.001^**^

VN, Vestibular neuritis; VSS, Vertigo symptom scale; DHI, Dizziness handicap inventory; HADS: The 14-item hospital anxiety and depression scale; VR, Vestibular rehabilitation; SE, Standard error; and OR, Odds ratio. ^*^p < 0.05, ^**^p < 0.01, and ^***^p < 0.001.

**Figure 3:**
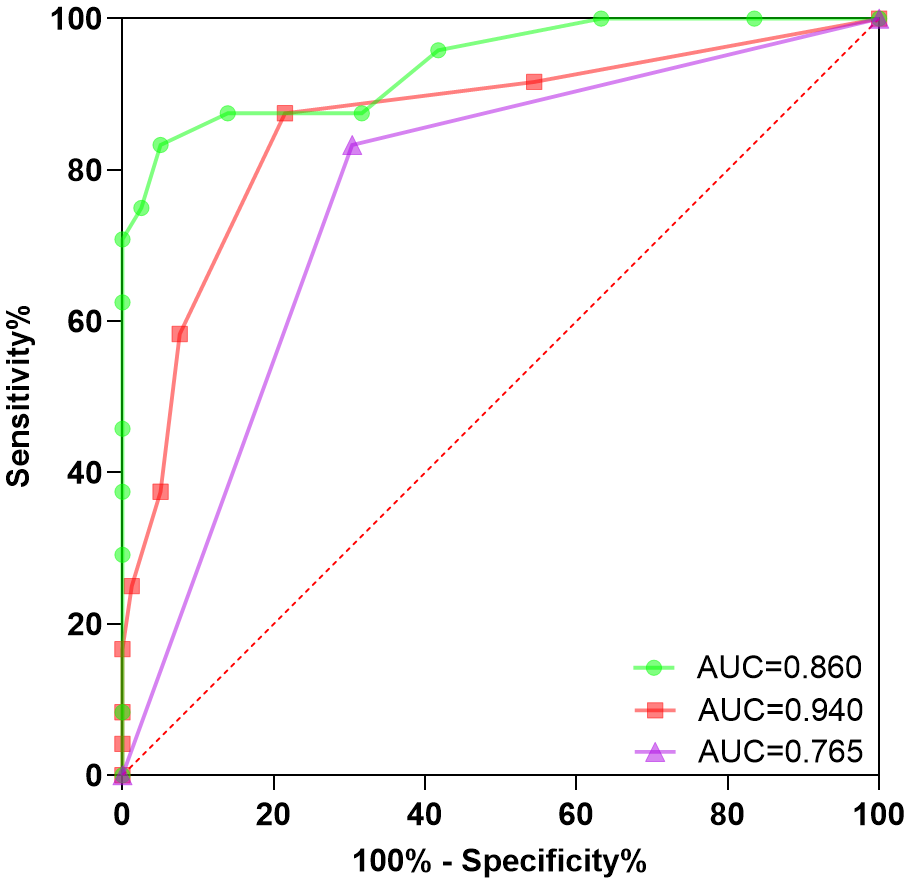
ROC curve for secondary PPPD prediction. This study conducted a rigorous evaluation of the predictive accuracy of a Receiver Operating Characteristic (ROC) curve in determining secondary PPPD. The findings provide valuable insights into the diagnostic potential of the ROC curve for identifying secondary PPPD in clinical settings, contributing to improved patient management. ROC, Receiver operating characteristic; PPPD, Persistent postural-perceptual dizziness; Green, DHI score at 6 months after onset; Red, Anxiety level at 6 months after onset; and Purple, Normalization of vestibular rehabilitation treatment.

We plotted ROC curves to predict PPPD in patients using different indicators. The results showed that anxiety level at 6 months after onset had the highest area under the curve (AUC = 0.940, 95% CI 0.881–0.999, *p* < 0.000, sensitivity 83.330%, specificity 94.940%), followed by DHI score at 6 months (AUC = 0.860, 95% CI 0.766–0.953, *p* < 0.000, sensitivity 87.500%, specificity 78.480%) and whether the patient received standardized vestibular rehabilitation therapy (AUC = 0.765, 95% CI 0.659–0.871, *p* < 0.000, sensitivity 83.330%, and specificity 69.620%). The ROC curves are shown in Figure 3.

## 4. Discussion

Our study found that the incidence of PPPD in the modular management group was significantly lower than that in the non-modular management group. Therefore, standardized “modular management” for VN patients will help improve prognosis, reduce the incidence of secondary PPPD, and minimize disease chronicity, thus promoting comprehensive and rapid recovery of social function in patients.

### 4.1. Health education and popularization

To further investigate the reasons for the reduction of chronicity by modular management, we analyzed the relevant clinical data of VN patients and found that initial misdiagnosis was an important risk factor for disease chronicity, but there was no clear correlation between the use of steroid therapy in the acute phase and the development of PPPD. Therefore, we believe that the difference in prognosis is not due to differences in acute drug treatment, but rather whether patients receive a clear diagnosis and disease-related education during the acute phase. Patients with an unclear diagnosis are more likely to develop anxiety, depression, and fear, leading to self-imposed physical restrictions such as reducing neck movement, slowing walking speed, shortening step length, and reducing joint movement during walking. These physical restrictions further lead to abnormal skeletal muscle function, ultimately limiting patients’ social activity ability at both physiological and psychological levels.

Patients in the modular management group received a clear diagnosis and disease education from specialist doctors at the earliest time, which can greatly relieve patient anxiety, enhance patient confidence in disease recovery, and take active coping measures, thereby reducing the occurrence of chronicization. Therefore, in clinical practice, active health education for vestibular neuritis patients, encouraging patients to actively remove restrictions, increasing active exercise and life scenario exposure, will help with patient recovery.

### 4.2. Whole-course VR therapy

McDonnell et al. (32) conducted a review of 39 trials involving over 2,400 patients with unilateral vestibular dysfunction, the authors concluded that there is moderate to strong evidence supporting the use of vestibular rehabilitation therapy (VRT) as a safe and effective treatment for unilateral peripheral vestibular dysfunction. Our study also confirmed the effectiveness of VR treatment, and the low utilization rate of standardized VRT was found to be associated with the occurrence of PPPD, indicating that standardized VRT may help prevent the chronicization of VN. The mechanism may be that early VRT can prevent the formation of poor posture, while head-eye coordinated movements can promote central compensation. In the recovery phase, VRT focuses on improving postural stability and reducing visual dependence, as the development of visual dependence is a major risk factor for chronicity in vestibular disorders (33) and a core symptom of PPPD (8). Physical therapy interventions have been demonstrated to decrease dizziness induced by visual stimuli (34).

### 4.3. Assessment of anxiety and depression

Additionally, our study found a significant correlation between patients’ poor prognosis and their anxiety and depression scores. Beck (35) and Bandura (36) point out the importance of the expected consequences of the event. They suggest that anxious individuals react more strongly to threatening stimuli and search more intensely for safety signals. This increases self-observation, which, in turn, tends to trigger negative emotions (37). Herdman et al. (38) found that approximately 75–88% of patients with unilateral vestibular dysfunction can benefit from VRT, but not all patients do, and anxiety and depression are the primary factors affecting rehabilitation outcomes. Cousins et al. (39) found that overactive autonomic nervous system and psychological factors are closely related to the development of visual dependence after unilateral vestibular damage. Compensation for unilateral vestibular loss depends on the reweighting of multisensory (visual-vestibular) cues, and the neural networks that process visual, vestibular, and emotional states are extensively linked and cross-connected. Thus, vestibular compensation and psychological states interact with each other, as supported by functional magnetic resonance imaging data, although the directionality of this association remains unclear (40). Therefore, we can infer that emotional disorders may interfere with VN recovery in various ways. Early identification and intervention to reduce anxiety and autonomic nervous system activation may have significant value in improving long-term prognosis. Comprehensive treatment plans, including patient education, CBT, and anti-anxiety/depression medication, may be beneficial for patients with moderate to severe anxiety or anxiety traits, potentially reducing the risk of chronicity.

## 5. Conclusion

We have found that some VN patients may experience recurrent episodes of vertigo during their recovery period, and even fully compensated patients may experience fluctuating vestibular symptoms and balance impairments. We need to carefully differentiate the different phenotypic combinations during different stages of the disease, explore the triggering factors and background diseases. The prognosis of VN may be related to multiple factors, and the weight of each factor may vary individually among patients. Early and accurate prediction of prognosis may facilitate personalized intervention for high-risk patients, preventing chronic disease and avoiding over-treatment.

However, our study has limitations such as small sample size, incomplete data on patients in the non-modular treatment group, and retrospective design. Future work will focus on finding sensitive and specific clinical indicators in the early stage of the disease, designing a clinical prediction model, and providing help for personalized treatment of early-stage disease.

## Data availability statement

The original contributions presented in the study are included in the article/supplementary material, further inquiries can be directed to the corresponding authors.

## Ethics statement

This study was approved by the Ethics Committee of Union Hospital, Tongji Medical College, Huazhong University of Science and Technology, Wuhan, China (NO. 20210873). All procedures performed in the studies involving human participants were in strict accordance with the ethical standards of the institutional and/or national research committee and with the 1964 Helsinki Declaration and its later amendments or comparable ethical standards.

## Author contributions

SZ and JZ designed the research and directed its implication. FL, JX, and DL prepared and analyzed the data and reviewed drafts of the manuscript. JW, LL, RG, and XZ contributed to the manuscript’s modifications. All authors contributed to the article and approved the submitted version.

## Funding

This work was supported by grants from the National Natural Science Foundation of China (Nos. 82171152 and 81873701), the Fundamental Research Funds for the Central Universities (HUST: YCJJ202201040), and Knowledge Innovation Program of Wuhan-Basic Research (No. 2022020801010458).

## Conflict of interest

The authors declare that the research was conducted in the absence of any commercial or financial relationships that could be construed as a potential conflict of interest.

## Publisher’s note

All claims expressed in this article are solely those of the authors and do not necessarily represent those of their affiliated organizations, or those of the publisher, the editors and the reviewers. Any product that may be evaluated in this article, or claim that may be made by its manufacturer, is not guaranteed or endorsed by the publisher.
